# Application of decision analytical models to diabetes in low- and middle-income countries: a systematic review

**DOI:** 10.1186/s12913-022-08820-7

**Published:** 2022-11-23

**Authors:** Tagoe Eunice Twumwaa, Nonvignon Justice, van Der Meer Robert, Megiddo Itamar

**Affiliations:** 1 Department of Management Science, University of Strathclyde, Glasgow, UK; 2 School of Public Health, University of Ghana, Legon, Ghana

**Keywords:** Decision analytical modelling, Diabetes, Economic evaluation, Simulation model, Cost-effectiveness

## Abstract

**Background:**

Decision analytical models (DAMs) are used to develop an evidence base for impact and health economic evaluations, including evaluating interventions to improve diabetes care and health services—an increasingly important area in low- and middle-income countries (LMICs), where the disease burden is high, health systems are weak, and resources are constrained. This study examines how DAMs–in particular, Markov, system dynamic, agent-based, discrete event simulation, and hybrid models–have been applied to investigate non-pharmacological population-based (NP) interventions and how to advance their adoption in diabetes research in LMICs.

**Methods:**

We systematically searched peer-reviewed articles published in English from inception to 8th August 2022 in PubMed, Cochrane, and the reference list of reviewed articles. Articles were summarised and appraised based on publication details, model design and processes, modelled interventions, and model limitations using the Health Economic Evaluation Reporting Standards (CHEERs) checklist.

**Results:**

Twenty-three articles were fully screened, and 17 met the inclusion criteria of this qualitative review. The majority of the included studies were Markov cohort (7, 41%) and microsimulation models (7, 41%) simulating non-pharmacological population-based diabetes interventions among Asian sub-populations (9, 53%). Eleven (65%) of the reviewed studies evaluated the cost-effectiveness of interventions, reporting the evaluation perspective and the time horizon used to track cost and effect. Few studies (6,35%) reported how they validated models against local data.

**Conclusions:**

Although DAMs have been increasingly applied in LMICs to evaluate interventions to control diabetes, there is a need to advance the use of DAMs to evaluate NP diabetes policy interventions in LMICs, particularly DAMs that use local research data. Moreover, the reporting of input data, calibration and validation that underlies DAMs of diabetes in LMICs needs to be more transparent and credible.

**Supplementary Information:**

The online version contains supplementary material available at 10.1186/s12913-022-08820-7.

## Background

The prevalence of diabetes, particularly type 2 diabetes, is rapidly increasing worldwide, particularly in low- and middle-income countries (LMICs). Around 537 million adults globally lived with diabetes in 2021, an increase of 70 million since 2019 [1]. The International Diabetes Federation (IDF) estimated that the number will increase by 46%, i.e., 783 million adults, by 2045 [1]. However, this projection may be underestimated considering the unintended effect of control strategies associated with multiple waves of Coronavirus Disease 2019 (COVID-19) on diabetes risk distribution, for example, increased stress and low physical activity levels due to lockdown [2]. Although 79% of persons with diabetes live in LMICs, total health expenditure on diabetes remains lower in LMICs than in high-income countries (HICs) because of resource constraints and lower priority given to chronic diseases [1, 3].

Additionally, diabetes accounts for a large proportion of morbidity and mortality in LMICs. IDF indicates that the Western Pacific and South-East countries experienced the highest diabetes fatalities between 2010 and 2019 [1]. Diabetes is one of the leading causes of death in LMICs among people aged 20–99 years, with the majority of deaths occurring in people under 60 years of age [1]. According to the IDF Atlas, Africa recorded more than a 10% increase in diabetes deaths between 2010 and 2019. Compared with deaths from other territories, most people who died in Africa were below 60 years old. South Africa and the Democratic Republic of Congo were the most affected by diabetes mortalities [1].

Studies have reported the potential of non-pharmacological (NP) population-based strategies to reduce the diabetes burden [4], but most of these studies are large-scale randomized control trials (RCTs) and cohort studies conducted in high-income countries, and transferability issues may limit the application of the evidence in LMICs. NP diabetes interventions are public health community-based strategies targeted at persons with or without diabetes to control or prevent the disease. They include fiscal policies, legislation in – but not limited to – trade and agriculture, health promotion activities or changes to physical environments that can influence modifiable risk factors, e.g., physical activity and dietary patterns. RCTs and cohort studies can generate evidence of the economic and health effects of NP diabetes interventions for policy decision-making [5], but their application can be challenging from an ethical, implementation time and cost perspective. Ethical issues, including – but not limited to – delaying life-saving intervention in human subjects, could constitute a breach of duty of care; consequently, ethics committees may deny such studies approval. In LMICs, large-scale RCTs and cohort studies may be challenging to fund.

Decision analytical models (DAMs) overcome these challenges by using mathematical and logical relationships to abstract real-world phenomena for investigation and simulation. DAMs are most useful where the lack of data due to a rare event, legal circumstances, time, technical, ethics and funds prevent real-live studies. LMICs can benefit from their use to prioritise interventions and policies as they enable comparatively cheap, convenient, and risk-free experiments that are impossible in reality [5]. DAMs can offer an opportunity to perform “what-if” scenarios to predict and explore NP diabetes interventions in LMICs before these interventions are implemented, saving policymakers the cost and time that would have been invested in trial and error.

Despite the benefits of DAMs, their application to diabetes in LMICs is limited. Most DAMs for type 2 diabetes are built among high-income populations and then generalized to LMICs, which could be misleading considering that the difference in culture, ethnicity and health system capacities influence diabetes control. For instance, diabetes develops 10 years earlier and at lower body weight in Africans and Asians than in Europeans [6]. There is a need to consolidate the literature on DAMs application, particularly their methodology, to diabetes in LMICs to identify gaps in their adoption and advance their use.

Whereas studies have appraised the application of DAMs to study diabetes interventions [7–11], few have focused on NP diabetes interventions in LMICs. Our study adds to this research. Mukonda, Cleary and Lesosky’s review on computer simulation models for type 2 diabetes in LMICs, like our present study, reports on model-based economic evaluations to support decisions in type 2 diabetes care, to assess their quality and validity [11]. However, unlike Mukonda and colleagues, we focus on DAMs for NP interventions and consider both type 1 and 2 diabetes. NP interventions are important for reducing diabetes risk and improving its outcomes [3], providing benefits to both diabetic and non-diabetic populations. Our review focuses on NP interventions as they are understudied in the literature compared to pharmacological interventions [11]. Moreover, the methods (and outcomes reported) used to investigate NP interventions can be very different from those used to examine pharmacological interventions as the latter concentrates on biology, physiology, and medicines compared to NP intervention, for which local context, including culture, health system and infrastructure is so important [3, 11]. As a result, it is often more reasonable to transfer efficacy estimates of pharmaceutical interventions than NP ones from RCTs in one setting to another. Thus, the methods for conceptualizing and developing models and the types of models that are fit for purpose differ for NP interventions. DAMs are also employed for scaling up pharmacological interventions’ use to understand the effectiveness of distributing these interventions in a population. The modelling focus and assumptions differ compared to NP ones. By focusing on NP interventions, we maintain a more homogenous set of studies and gain insight into this understudied area.

Specifically, our review investigates the methodological application of DAMs investigating NP diabetes interventions in LMICs with three objectives:

1. To investigate how DAMs have been applied to investigate NP policy interventions in LMICs,
2. To assess what gaps exist in the modelling procedures according to the Consolidated Health Economic Evaluation Reporting Standards (CHEERS) checklist.
3. and how to advance their adoption to diabetes research in LMICs.

We focus on four types of DAMs: systems dynamic, agent-based models, microsimulations, discrete event simulations and Markov models and a hybrid of any of them, hereafter referred to as DAMs, in NP diabetes intervention. Multiple factors determine the choice of DAMs in a study, e.g., study problem and goal, system processes, data availability and aggregation level, and analysis unit. Each approach has specific assumptions and structure, detailed in Additional file 1.

## Methods

We qualitatively review peer-reviewed articles that examine DAMs of the natural progressions of type 1 and 2 diabetes and assess the effect of NP interventions on these populations. The qualitative review was designed and presented using Preferred Reporting Items for Systematic Reviews and Meta-Analyses (PRISMA) guidelines [12].

### Information sources and search strategies

A combination of search terms for diabetes and the four modelling approaches (indicated below) was used to search titles and abstracts of papers in PubMed and Cochrane databases from inception to 8th August 2022. We limited the results to peer-reviewed articles published in English. Two people were involved independently in the search strategy phase, and disagreements were resolved through discussions and third-party judgement.

(((diabetes) OR (type 1) OR (type 2) OR (NIDDM) OR (IDDM) OR (DM)) AND ((Diabetes Mellitus [MeSH Terms]))) AND (“agent-based” OR “system dynamic” OR “discrete event” OR “microsimulation” OR “Markov model”)

### Eligibility criteria

Table 1 summarises the inclusion criteria applied. We included studies that meet the following criteria: 1) use any of the four DAMs or a hybrid of them, 2) examine NP interventions among persons living with type 1 or 2 diabetes or at risk of type 2 diabetes (as defined in the papers), 3) conducted the study in LMICs population per World Bank 2020 classification or initialized the modelled population with data from LMICs.

**Table 1:** Inclusion and exclusion criteria applied in the study

Parameter	Inclusion criteria	Exclusion criteria
Population	Type 1 and 2 diabetes patients LMICs.	Gestational diabetes, non-diabetics Diabetes is secondary to a primary condition, e.g., diabetes in schizophrenia patients.
Intervention	DAMs that examine non-pharmacological population-based diabetes interventions for type 1 and 2 diabetes.	Medicine, medical technology, and medical procedure-related interventions. Adopts an existing developed model.
Study design	Markov, microsimulation, system dynamics, discrete-event, agent-based modelling studies with a component of economic evaluation: benefit, cost, or cost-effectiveness analysis.	
Evidence / Time frame	Peer-reviewed articles Published in English From inception to 8th August 2022.	Models that examine or advance methodological approaches

*LMICs* low- and middle-income countries, *DAMs* decision analytical models

### Exclusion criteria

Studies were excluded where 1) diabetes control is secondary to a primary disease/medical condition, e.g., preventing diabetes in people with schizophrenia, because in such studies, the model is designed with a particular focus on the primary disease and 2) they focus on pharmaceutical interventions and the pathological disposition of diabetes. Studies were excluded where the data, i.e., diabetes prevalence, obesity, and blood glucose distribution—used to initialize the modelled population were from developed countries. Diabetes prevalence and risk are higher in most developed countries; therefore, using data from such countries would likely misrepresent the disease burden in LMICs. All qualitative studies, literature reviews, abstracts only, non-English written papers and methodological papers were excluded. For instance, we excluded Kazemian et al.’s study [13] titled “development and validation of PREDICT-DM: a new microsimulation model to project and evaluate complications and treatment of type 2 diabetes mellitus” because the paper focused on the modelling process, i.e., the design, development, and validation of the model for the United States population.

### Data collection process

We used a data extraction tool containing relevant information from the methods section of the CHEERS checklist [14] needed to answer the research question to collect data from selected articles. The checklist is recommended to standardise and improve the reporting of economic evaluation of health interventions; it provides guidelines for model-based economic evaluation. We then summarised data under categories using Nvivo 12.0 and presented the summary in an excel format. The summarised data from selected articles are in Additional file 2.

### Data items

Pieces of information were extracted from the selected articles and organized under themes to answer the study questions. We collected and organized data under five themes: “study details”, which included data items such as author(s), title, publication date and place; “diabetes details”, consisting of the following data items: the type and diabetes state modelled, interventions and outcomes reported. “Model design” included model choice, parameterization and calibration, uncertainty analysis and validation. “Limitations” gathered information on study limitations reported in reviewed publications, and “economic evaluation” gathered details on the type of evaluation conducted, perspective adopted, interventions assessed, and outcomes reported.

## Results

### Study selection

**Fig. 1:**
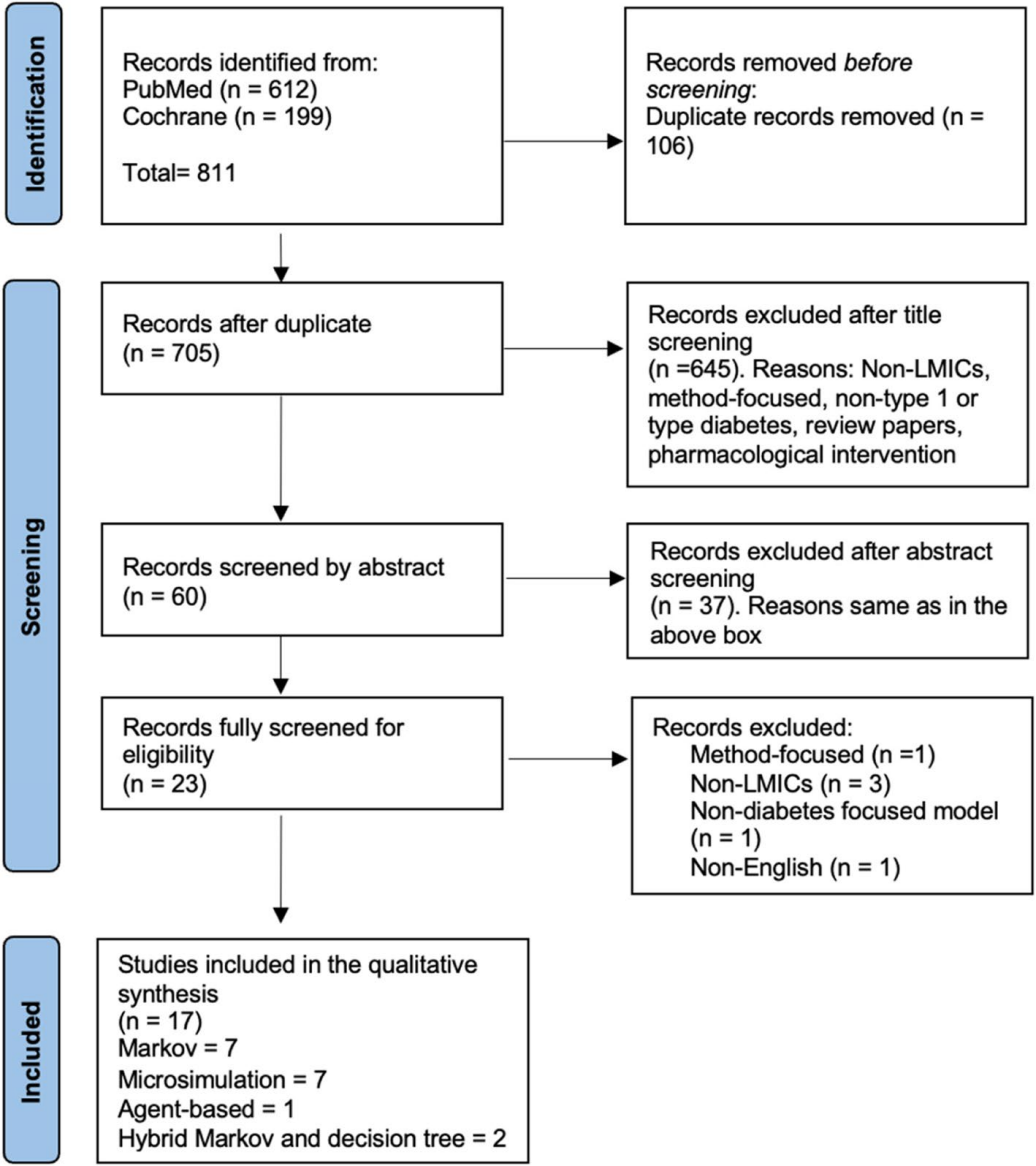
Article selection process

Figure 1 highlights the process of article identification, screening, and selection. Our initial search resulted in 811 articles, and no relevant article was retrieved from searching the reference list of selected articles. Overall, we screened the full text of 23 studies and then included 17 studies in the qualitative review. Table 2 summarises findings from reviewed studies. Details of included studies are in Additional file 2. Studies excluded in the full-text screen and the justification are in Additional file 3, as well as the PRISMA checklist in Additional file 4.

**Table 2:** Modelling details in CHEERs checklist reported in reviewed studies

**Model Design**	***N*, % (*N*** **= 17 studies)**
*Model type*	
Markov	7, 41%
Microsimulation	7, 41%
Agent-based	1, 6%
Hybrid Markov and decision tree	2, 12%
*Parameter uncertainty*	
One-way	4, 24%
Multiway	1, 6%
PSA^a^	4, 24%
Multiple strategies	7, 41%
Not reported	1, 6%
*Intervention(s)*	
Fiscal policy	3, 18%
Screening	5, 29%
Health system operations	2, 12%
Patient education	3, 18%
Not specific^b^	4, 24%
*Validation*	
Reported	6, 35%
Not reported	11, 64%
**Economic evaluation**	*N*, % (*N* = 11 studies)
*Outcomes*	
Benefits only	4, 24%
Cost only	2, 12%
Cost and benefit	11, 65%
*Perspectives*	
Societal	5, 45%
Health system/provider	4, 36%
Both	2, 18%
*Time horizon*^*c*^	
> =25 years	6, 55%
26–50 years	4, 36%
Lifetime	1, 9%
*Discount*^*c*^	
Cost only	2, 18%
Effect only	0, 0%
Both cost and effect	9, 81%

^a^*PSA* Probability sensitivity analysis

^b^Not specific means that intervention modelled was broad, did not specify activities policymakers could implement, e.g., reduction in obesity prevalence, and increasing dairy food consumption compared to screening for diabetes

^c^among studies that conducted a cost-effectiveness analysis

### Country and year of research

The majority of the modelled population was from China (5 studies) and India (4 studies). Two studies each described sub-populations in LMICs and Palestine, whereas one study each modelled sub-populations in Iran, Cambodia, South Africa, and Brazil. Studies were published between 2013 and 2021, with the highest number of publications (eleven) occurring between 2016 and 2020.

### The type and diabetes progression state modelled

Nine studies (69%) [15–23] modelled adults at risk of diabetes and undiagnosed diabetes; one of the nine studies specifically looked at persons with impaired glucose tolerance [21]. The remaining eight studies [24–31] modelled persons with diagnosed diabetes (type 1 or 2 or both) under treatment.

### Interventions modelled/strategies studied

Screening programs were the most common intervention examined, as they were reported in five studies [18, 19, 23, 27, 29]. Screening interventions targeted at-risk populations except for two studies [27, 29] that investigated diabetes retinopathy screening strategies among diabetes patients aged 40 years and above under clinical treatment. Three studies each investigated interventions related to fiscal policies [16, 22, 28] and patient education [21, 24, 25]. Specific interventions under the formal include taxes on sugar-sweetened beverages [16], food aid [22] and financial coverage for clinical diabetes care [28]. Specific patient education-related interventions reported include lifestyle-focused planned diabetes programs [24, 25] and the delivery of short message services on diabetes care [21]. An additional two studies (46%) investigated interventions related to health systems’ operations: one on clinicians’ choice of treatment approaches [26] and the other on national/international treatment guidelines [31]. As shown in Table 2, four studies do not describe the specific interventions but broadly describe the interventions as obesity reduction [15], dairy food consumption [17], healthy lifestyle behaviours [20] and increasing diagnosis, treatment, and control [30], but these studies do not describe the activities/program that would reduce obesity, increase dairy food consumption promotes a healthy lifestyle and improve treatment/control. For instance, Abu-Rmeileh et al. [15] assume a 5, 10, 15 and 35% reduction in obesity prevalence and model the outcome of such reductions on diabetes prevalence in Palestine but do not describe measures to be taken to achieve the reduction.

### Decision model type

Markov models and microsimulation models (seven studies each) were the most used modelling approaches, as indicated in Table 2. Only one study [20] used an agent-based model (ABM) to examine lifestyle intervention for diabetes prevention in China. No study was found that used discrete event simulation (DES) and system dynamics (SD) models. The review found no study that combined a Markov model, SD, DES, microsimulation, or ABM, except for two studies that combined a Markov model with a decision tree—a series of nodes and branches representing a logical structure of possible decisions and resulting [5].

### Benefits of decision modelling

Only two studies (15%) indicated the benefits of using DAMs [21, 24]. Both studies indicated time, cost, ethical considerations, and non-practicality of conducting long-term clinical trials as justification. Another explanation provided is the complexity of diabetes. The disease progresses gradually over a long time, and it is associated with several complications and other diseases that could be difficult to represent in a real-world experiment [24].

Seven studies discussed the benefits of using the specific modelling approach used. Three studies mentioned that the benefit of microsimulation over Markov models was mainly to represent complex relationships, individual-level dynamics, and histories [18, 26, 31]. One study [20] that investigated how individual-level factors influence public health outcomes found ABM beneficial in capturing interactions and feedback between individual-level behaviour and population-level parameters.

### Software

All but three studies, each using ABM [20], microsimulation [30] and hybrid Markov and decision tree [23], indicated the software used to implement models. Six studies used TreeAge software, out of which four were used to implement Markov models and one each for microsimulation and hybrid Markov and decision tree. An additional five studies used R software (four microsimulations and one Markov model). MATLAB was used to implement microsimulation [16] and Markov models [24]. One study used Microsoft Excel to implement a Markov model [15]. Three studies did not report on the software package used for modelling.

### Uncertainty analysis

Uncertainty analysis examines the accuracy of model outcomes and how model outcome changes due to variations in model parameters, structure, or assumptions [5]. Modellers may perform first-order, second-order, or structural uncertainty analysis to investigate uncertainty. The former investigates uncertainty surrounding outcomes for individuals with the same characteristics and cannot be conducted in deterministic models. Second-order examines uncertainty around model parameters, and structural uncertainty investigates other uncertainties that do not fit directly into parameters, methodological or heterogeneity [5].

Techniques for investigating second-order uncertainty are one-way, multiway/threshold and probabilistic sensitivity analysis (PSA) [5]. In a one-way sensitivity analysis, a single parameter or assumption is varied at a time, whereas in multiway, parameters or assumptions are varied simultaneously to the highest or lowest values to generate a best- or worst-case scenario; both methods are deterministic. In PSA, a model’s parameters are sampled from pre-specified distributions. The outcome of PSA is an interval within which model outcomes could fall. Table 2 shows the distribution of techniques studies used in parameter uncertainty analysis. All reviewed studies except one [24] indicated the methods used to estimate parameter uncertainty. Seven studies (41%) used several methods: five combined one-way and PSA [16, 18, 26, 27, 29], one combined one-way and multiway analysis [21] and one combined one-way, multiway and PSA [23]. Nine studies (54%) used a single method: four each used one-way only [19, 20, 25, 30] and PSA only [17, 22, 28, 31] and one used multiway analysis only [15].

### Validation/confidence building

Validation is the process of comparing model components, e.g., structure, input, outcome, and assumptions, with reality to increase confidence in the model outcomes. Validation techniques include face (expert assess model behaviour and processes), internal/verification (check coding accuracy), cross (compare model outputs with outputs of similar models), external (compare model output with actual data) and predictive validity (compare model output with prospectively observed data). Multiple techniques can be used to increase confidence in models.

More than half of the reviewed studies (11 studies, 45%) do not report model validation procedures. Model users and consumers of model results/outputs may have less confidence in unvalidated models and are less likely to use such models or their outcomes [32]. Among the six studies (46%) that reported validating their models, four conducted external validity using historical data from national surveys and literature review [15, 16, 18, 27]. The remaining two studies [17, 20] combined all four validation techniques: 1) face validity through expert consultation, 2) internal validity through sensitivity analysis and manually checking codes for error, 3) cross validity via comparing model outputs to outputs of similar published models, and 4) external validity using actual data from national/international surveys registers.

### Data source, parameter estimation and calibration

Data may be unavailable or scattered in different sources and forms that are not readily usable in a model, requiring modellers to combine or transform available data through mathematical methods to estimate and calibrate model parameters. Reviewed studies obtained data to parameterized models from plausible assumptions, previous simulation studies, published cohort studies and data recorded in clinical trials, national surveys, and registers. Except for one study [24], which used primary data from a diabetes preventions program, all studies reported data limitation as a challenge to model building. Researchers reported that local epidemiological and clinical data (e.g., diabetes state transition probabilities and risk equations) and cost data were mostly unavailable, unreliable, or insufficient for modelling purpose, consequently leading them to use data from international studies. Studies used mixed-effects meta-regression, inverse-variance methods, validated hazard calculation method and validated equations from the United Kingdom Prospective Diabetes Study, Risk Equations for Complications of Type 2 Diabetes, World Health Organisation (WHO) and International Society of Hypertension equations, and Monte Carlo Markov Chain to parameterized and calibrate models.

### Type of evaluation (health and economic)

Economic evaluation can produce evidence of the cost and the effect of alternative interventions to support decisions on resource allocation. Among the reviewed studies, four reported on health effects only [15, 16, 20, 24], and two reported on cost/economic outcomes only [17, 18] from a health system perspective. Eleven studies (54%), whose details are presented in Table 2, included evidence of both cost and health outcomes. For instance, Liu et al. [19] examined the cost of screening and lifestyle interventions from a societal perspective and the resulting health effects, including life-years remaining, quality-adjusted life years (QALYs) and incremental QALYs.

## Discussion

The chronic nature of type 1 and 2 diabetes, high cost and implementation challenges of large-scale cohort studies necessitate the use of decision analytical modelling to extrapolate evidence of short-term empirical studies to predict costs and health benefits over a long-term period. This study reviewed the application of DAMs, specifically Markov cohort models, system dynamics, discrete event, microsimulation, and agent-based models, to study NP diabetes intervention in LMICs. We applied the CHEERS checklist to appraise seventeen studies identified through a systematic search.

Overall, there were more studies conducted in Asian sub-populations, particularly China, and most studies reported data as a limitation. The dearth of studies exclusively from LMICs is somewhat indicative of the scarcity of the use of DAMs in research in the region. Healthcare data sources exist in LMICs, e.g., national demographic and health surveys and WHO’s studies on Global Aging and Adult Health. However, the levels of detail and quality are often insufficient. Consequently, confidence in the validity of outcomes/results from models using such data sources could be questioned. This is daunting given the expected escalation of the diabetes burden, and it highlights the need for more structures for data collection/generation in LMICs.

In the absence of sufficient and quality data in LMICs, calibration techniques are used to adjust parameters or even existing models with different population characteristics, e.g., models from HICs. The objective of (re)calibration is to enhance the representativeness or predictability of a model which otherwise might under- or over-estimate model outputs. Calibration approaches reported in the reviewed studies include Markov Chain Monte Carlo —a standard Bayesian posterior computation approach [33] and adjusting the Risk Equations for Complications of Type 2 Diabetes (RECODe) with local data. RECODe was designed for Americans in particular; however, the equation can be adjusted with local data to represent context-specific epidemiology.

The International Society for Pharmacoeconomics and Outcomes Research have also produced protocols for the adaption of an existing validated model to a specific context/ population [34]. During such an adaption process, it is essential that baseline characteristics of the modelled cohort are adjusted to reflect local epidemiological data and possibly the transition probabilities between states. Also crucial for the adaption are context-specific cost, health state preferences, and utility data. The requirement of context-specific data brings us back to the challenge of reliable/robust diabetes data in LIMCs and how the situation limits the application of DAMs for diabetes in the region. Modellers could obtain model data from various sources: ongoing clinical trials or existing data through systematic review and meta-analysis, routine data collection, expert opinion, and observational studies. Among these data sources, researchers have suggested using observational research data on type 2 diabetes in LMICs despite concerns about selection bias as compared to RCTs, highlighting how such metrics, including health-related quality of life, are seldom altered by selection bias [35]. As a result, observational studies, which are easier to conduct in LMICs, can fill in the data gaps required for the implementation of DAMs.

Model validation is another procedure to improve models’ fitness for purpose, especially when adapting parameters or existing models developed from other populations. Nearly half of the reviewed publications do not report how models were validated, and few discussed how applicable their model is in other LMICs. Some of the reviewed studies mentioned using data from the United Kingdom Prospective Diabetes Study (UKPDS) Outcomes Model and RECODe to estimate transition probabilities and risk equations for predicting diabetes complications. It is worth noting that both were developed for the United Kingdom and United States populations, respectively. Even though these models had undergone external validation, it is unclear if the models had also been validated for the LMIC populations of interest.

Tarride and colleagues [36] have identified some drawbacks to using UKPDS data: 1) the majority of UKPDS participants were of European descent and considering that diabetes progresses differently in different ethnic groups, there is a challenge with generalizing data to varied ethnic groups. 2) Technology and treatment regimens have advanced since the study was conducted (1977–1997). Such developments, e.g., new medicines and disease incidence, could affect the transition between diabetes states, affecting the applicability of the model. 3) Differences in culture, lifestyle, demographics, health systems and available health technology means that the rate of diabetes progression in a United Kingdom population is different from LMIC populations, affecting the application of the model to LMICs. These issues highlight the need for extensive model validation and reporting on the validation process to increase model representativeness, transparency and confidence in model processes and outcomes, consequently increasing model adoption. Researchers could adopt different tools, e.g., CHEERs checklist, International Decision Support Initiative (iDSI) Reference Case for Economic Evaluation [37], Diabetes Modelling Input Checklist [38] and the Overview, Design concept and Details protocol [39] (in ABMs) to improve model reporting and transparency.

Although most of the reviewed studies used a Markov cohort model or microsimulation (Table 2), it is unclear what modelling approach is appropriate as their appropriateness depends on multiple factors, including the study question/objective. The assumption of “memoryless” property, meaning that transition to another state is independent of the previous states, is a fundamental characteristic of Markov modelling but also a drawback [5] as this would be a simplification for diabetes where progression to another diabetes state is dependent on the previous state. Given the substantial patient variability and interconnected risk in diabetes, complexity is almost unavoidable [9]. Another challenge with using Markov cohort models in a cost-effectiveness analysis is the likelihood of skewed estimates of the incremental cost-effectiveness ratio resulting from “uncaptured” patient heterogeneity [40].

Unlike Markov cohort models, microsimulation models individual characteristics through time, thereby representing patient variability and overcoming the Markov assumption. Individual level characteristics can affect how they move through the model and can also be utilized to modify the likelihood of future occurrences. Details of DAM approaches are available in Additional file 1. Microsimulation allows for modellers to assign risk of events or complications, taking into consideration a patient’s unique demographic characteristics, risk factors, and event history and is not constrained to “mutually exclusive” health states as is the case in Markov-based models. However, the challenge with microsimulation compared with a Markov cohort model is the heavy computation and data required [9]. In addition to data challenges, LMICs currently lack the technical capacity for building heavy computational models. Collaborations between international and local academic and research institutions could help increase modelling expertise in LMICs. Knowledge exchange initiatives, such as the Mount Hood Diabetes Challenge Network [38], which facilitates the sharing of ideas and skills among diabetes simulation modellers through workshops, hackathons, and conferences, can build capacity in LMICs for advancing DAMs.

Additionally, incorporating evidence of intervention cost and effect in DAMs can increase the use of such models as decision support in LMICs. Economic evaluation methods that use DAM estimate the potential lifetime impact of investments, which means policymakers can account for or justify their choices. Having such evidence can contribute to policymakers prioritizing diabetes interventions. The CHEERS protocol recommends reporting perspectives of economic evaluations and a lifetime horizon for tracking cost and effect. It is encouraging to note that all reviewed studies reported on the perspectives of their economic analysis, giving readers a context within which cost is tracked.

### Study limitations and directions for future research

The study’s main limitation is that research that would have added to the discussion may have been left out due to exclusion criteria, such as eliminating published material that is not presented in English or studies on pharmacological interventions. We eliminated the latter due to their difference in modelling methodologies, assumptions and focus compared to NP intervention studies. By collecting data relevant to modelling, important insights into the effectiveness and cost-effectiveness of the interventions being simulated are not reported. Further research should thoroughly analyze DAMs in connection to the decision issue at hand and the selection of cost-effective NP interventions. Second, although we judge the CHEERS checklist appropriate for this review, the Diabetes Modelling Input Checklist designed for improving transparency in reporting input data of model-based economic evaluation and the IDSI Reference case are equally suitable and should be explored by future studies. Nevertheless, given the large overlap with both checklists, we believe nothing significant has been overlooked that may have altered the findings of this analysis.

## Conclusion and implications

DAMs have been increasingly applied in LMICs to evaluate interventions to control diabetes, which may contribute to better decision-making about the best use of limited resources and the improvement of patient outcomes. However, there is a need to advance the use of DAMs to evaluate NP diabetes policy interventions in LMICs, particularly DAMs that use local research data, because 1) such models are a good representation of diabetes burden in LMICs, 2) and could be effective at estimating intervention effect on local population. Additionally, given that data limitation is a major concern in model-based assessments, especially in non-Asian LMICs, clinical and observational research on diabetes is undoubtedly necessary. Finally, the reporting of input data, calibration and validation that underlies DAMs of diabetes in LMICs needs to be more transparent and credible. Modellers are encouraged to use the CHEERS checklist and Diabetes Modelling Input checklist to improve the modelling process and documentation.

## Supplementary Information

**Additional file 1.** Description of Decision Analysis models.

**Additional file 2.** Details of reviewed studies.

**Additional file 3.** Characteristics of studies excluded during full text review.

**Additional file 4.** PRISMA checklist of the study.

## Data Availability

All data generated or analysed during this study are included in this published article [see Additional files].

## Abbreviations

LMICs: Low- and middle-income countries
DAMs: Decision analytical modelling approaches
NP: Non-pharmacological
ABM: Agent-based models
DES: Discrete event simulation
SD: System dynamics
NCD: Non-communicable diseases
QALY: Quality-adjusted life years
RCT: Randomised control trials
PSA: Probabilistic sensitivity analysis
HICs: High-income countries
RECODe: Risk Equations for Complications of Type 2 Diabetes
UKPDS: United Kingdom Prospective Diabetes Study (UKPDS)
CHEERS: Consolidated Health Economic Evaluation Reporting Standards
IDSI: International Decision Support Initiative
WHO: World Health Organisation
