# Supplementary material for: Application of decision analytical models to diabetes in low- and middle-income countries: a systematic review
Source: BMC Health Serv Res. 2022 Nov 23;22:1397. doi: 10.1186/s12913-022-08820-7 (PMC9684986; doi:10.1186/s12913-022-08820-7)
Supplement: Supplementary file 1 — Additional file 1. Description of Decision Analysis models. [file 12913_2022_8820_MOESM1_ESM.docx]

**Additional file 1**

**Description of Decision Analysis models**

*Markov Models*

Markov cohort model, Monte Carlo, and fundamental matrix are different Markov modelling approaches. Monte Carlo uses probabilities and random numbers to simulate the pathway for several persons starting at a particular state. In a fundamental matrix, transition probabilities and utilities/costs associated with all states remain constant; hence expected utility/cost could be calculated with a matrix (1). This study focuses on Markov cohort models, commonly used in healthcare decision analysis. Markov cohort models represent the pathway of an average person from a population with similar characteristics rather than representing individual-level variability. Probabilities are assigned to events or a percentage of the modelled cohort expected to experience event(s) to model transition between states. The consequences of being in a state, also called utilities or cost, are assigned to each state. Time is modelled in cycles—equal time intervals accumulating to the overall time horizon considered for analysis (1). At initialization, the hypothetical population is distributed among starting states, e.g., controlled diabetes and uncontrolled diabetes. During the simulation, individuals in a particular state are divided into other states by transition probabilities resulting in a new distribution for the next cycle (1). The assumption of “memoryless” property, meaning that transition to another state is independent of the previous states, restricts their application (2), as this would be a simplification for diabetes where progression to another diabetes state is dependent on the previous state.

*System Dynamic Simulation*

SD uses systems’ processes and feedback loops to investigate the complex interaction and effect of structure, policies, and time delays on organisational success (3). SD is a top-down approach to analysing complex systems through buildups and feedback, usually displayed in causal loop diagrams, to determine system structure and behaviour. In a top-down approach, a group of entities’ behaviour is assumed from the onset and then modelled ‘as a whole as opposed to bottom-up approaches, where group behaviour emerges from individual-level interactions (3). A causal loop diagram (collection of a system’s feedback loops) is a visual representation of system structure and a qualitative assessment of system behaviour. Causal loops are transformed into a stock-flow diagram to quantitatively determine the net effect of feedback loops on system behaviour. Stocks (buildups of flow rates due to differences in inflows and outflow) and flows (quantities added or subtracted from stock) are building blocks that describe a system’s state at a time. SD assumes continuous variables and models in continuous time (3).

*Discrete-event Simulation*

DES focuses on changes in a system state triggered by the succession of distinct events over time. Thus, DES is built on queues/queueing systems. Entities are static or dynamic objects, explicitly defined and managed through process listing—allocating them to resources that perform activities or provide services (4). DES comprises these concepts: work items, resources, routing, buffer, scheduling, sequencing, and performance (4). *Work items* represent individuals/items who enter the modelled system to seek services, e.g., diabetes patients (work) accessing outpatient care (services/activities). *Resources* are materials or human resources required for producing services. In the case of the preceding example, healthcare providers and medical equipment are resources. Healthcare resources are required for each diabetes patient (work item) seeking service(s), and services are delivered in order. The collection of services and the ordering of their performance is termed routing. *Buffer* is a finite or infinite list of works pending services. *Scheduling* describes patterns of resource availability, e.g., the number of doctors on a roster per month. When a queue/waiting list is countless, sequencing describes how limited resources provide services, e.g., using rules such as first-come-first-service and elderly persons first. DES can model open- or closed-loop systems. In open-loop systems, work emanates from outside the system, and their arrival time is unknown and uncontrolled by the modelled system. The opposite is true for closed-loop systems (4).

*Microsimulation Modeling*

Microsimulation models from an individual level using a bottom-up approach. Microsimulation modelling (MSM) is like Agent-Based Modelling (ABM) in that both techniques use a bottom-up approach to model interactions and simulate transitions between states (5). When MSM is run, individual entities are run through mechanistic processes such as a diabetes progression pathway. Entities in MSM and ABM could be heterogeneous with distinct characteristics, and in both modelling approaches, individual entities can interact (5). MSM can represent entities’ behaviour by abstracting from a pool of behaviour, but the entities themselves cannot make autonomous decisions.

*Agent-Based Modeling*

ABM is a bottom-up modelling approach characterised by system behaviour and patterns emerging from interactive and autonomous agents’ decisions. Typically, an ABM system consists of the environment, agents, and interactions among agents and between agents and the environment. Agents can be single or grouped autonomous and heterogeneous entities, e.g., individuals, communities, and countries, whose behaviours are defined by rules and commands (6). Thus, ABM can abstract reality from micro-, mezzo- or macro-level, depending on the study problem. A transition between states is modelled from a bottom-up approach using rules, inferences, and probabilities (6). Unlike MSM, where aggregation of individual characteristics amounts to the population characteristic, individual-level attributes may not sum up to the population/system-level features in ABM since some characteristics/behaviours emerge from non-linear interactions between autonomous agents and among entities.

There are currently diverse views on the definition of ABM and no clear distinction between MSM and ABM in the modelling literature. There is consensus that both approaches model bottom-up, micro-level, and multiple agents. However, it is unclear how both approaches differ in features and capabilities. Macal (7) identifies four definitions of ABM. Briefly and in no order: definition 1) emphasizes individual agents with different characteristics, 2) autonomous individual agents who can react to environmental stimuli, 3) individual agents can interact among themselves and with their environment and 4) autonomous and interactive individual agents learn and adapt to changes in their environment during simulation. In the context of this review, we consider ABM as models with heterogeneous and autonomous/intelligent agents who can interact with other agents and their environment. Table 1 summarises the main difference among the DAMs considered in this study.

Characteristics of decision analytical models

***
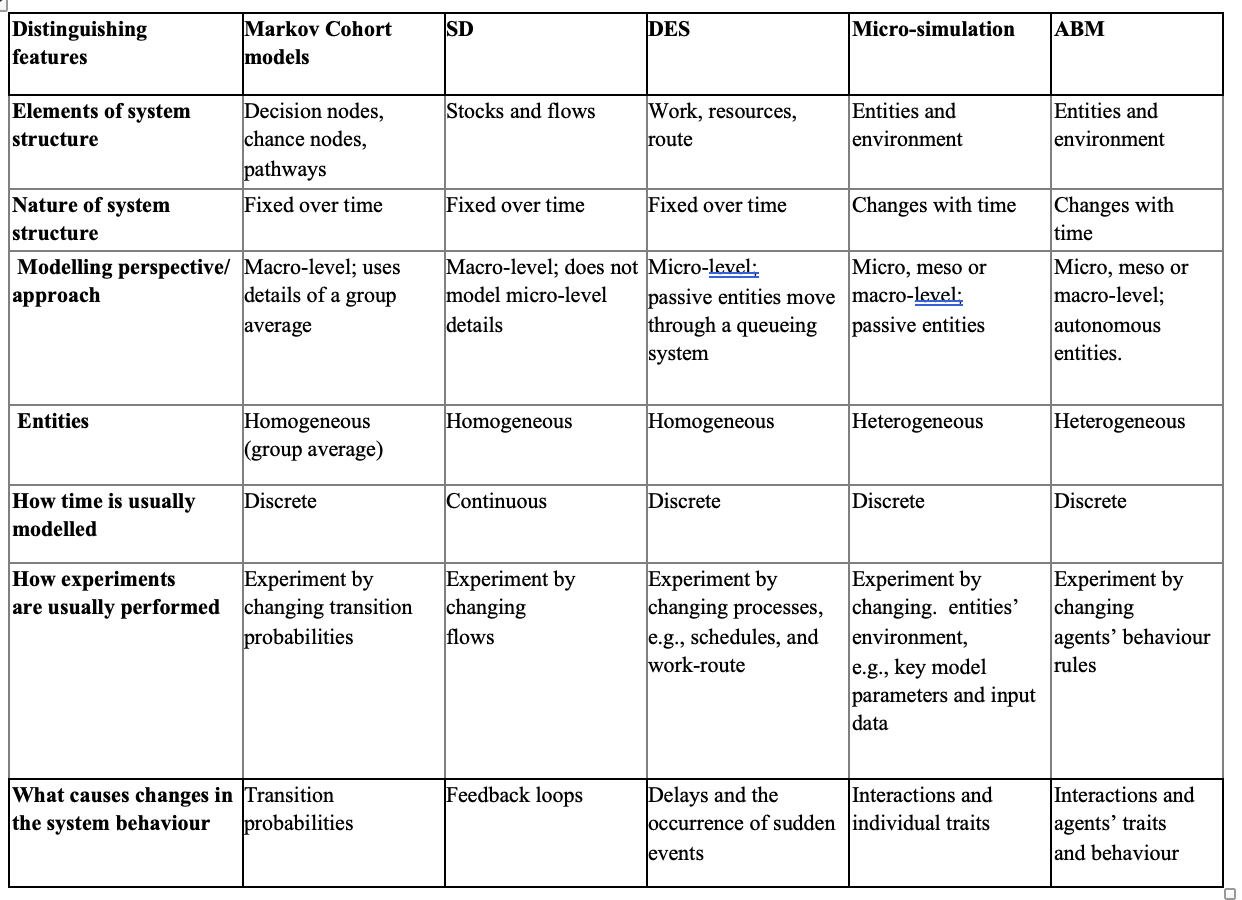
***

***Hybrid Simulation Designs***

Hybrid models combine multiple modelling methods to describe complex systems. For example, combining ABM and SD gives both a bottom-up and top-down paradigm for simulating complex adaptive systems. Morgan et al. (8) and Swinerd and Naught (9) proposed mixed method designs for hybridising DES and SD in particular but could be used to combine other models.

Hybrid model design types

| **Swinerd and Naught hybrid model design** | **Model Description/Explanation** | **Morgan et al. hybrid model designs** |
| --- | --- | --- |
| **Integrated** | A primary model is used as the base model, and then elements of a second model are used to enrich the primary model. The model is built as a single unit, and the problem dictates the need to incorporate elements of a second model.  With this description, Swinerd and Naught identify two forms of SD and ABM hybrid models designs:   1. Rich internally structured agent: SD model built within an ABM agent. E.g., Schieritz and Grobler  (10) 2. Stock agent: a component in SD is used to influence an aggregate parameter in ABM.   E.g., Verburg and Overmars (11) | **Enrichment** |
|  | Elements from two or more modelling approaches are combined and to form a new model. Interaction between the two models occurs at a fixed time step, e.g., Chaim and Streit (12) | **Integration** |
| **Interfaced** | Two or more modelling approaches from different paradigms are combined while relaxing paradigm restrictions. For instance, an ABM aggregate measure is used to influence parameter(s) in SD. E.g., Dubiel and Tsimhoni  (13) | **Interaction** |
| **Sequential** | A first modelling approach informs a second modelling approach; approaches complement each other. Both models run separately, and information is exchanged between them in consecutive runs. | **Sequential** |
|  | Multiple modelling approaches produce two potential representations of the same system, which then allows for comparison. | **Parallel** |

Note: Model descriptions/explanations match the model designs on the left (Swinerd and Naught’s hybrid designs) and right (Morgan et al.’s hybrid designs). Enrichment and integration designs by Morgan et al constitute Swinerd and Naught’s Integrated design. Morgan et al’s Parallel design has no corresponding design in Swinerd and Naught’s hybrid designs.

Reference:

1. Sonnenberg FA, Beck JR. Markov models in medical decision making: a practical guide. Medical decision making. 1993;13(4):322-38.

2. Briggs A, Sculpher M, Claxton K. Decision modelling for health economic evaluation: Oup Oxford; 2006.

3. Sterman JD. System Dynamics Modeling: Tools for Learning in a Complex World. California Management Review. 2001;43(4):8-25.

4. Fishman GS. Discrete-event simulation: modeling, programming, and analysis: Springer Science & Business Media; 2013.

5. Bae JW, Paik E, Kim K, Singh K, Sajjad M. Combining microsimulation and agent-based model for micro-level population dynamics. Procedia Computer Science. 2016;80:507-17.

6. Railsback SF. Agent-based and individual-based modeling : a practical introduction. Second edition.. ed. Grimm V, editor: Princeton ; Oxford : Princeton University Press; 2019.

7. Macal CM. Everything you need to know about agent-based modelling and simulation. Journal of Simulation. 2016;10(2):144-56.

8. Morgan JS, Howick S, Belton V. A toolkit of designs for mixing discrete event simulation and system dynamics. European Journal of Operational Research. 2017;257(3):907-18.

9. Swinerd C, Mcnaught KR. Comparing a simulation model with various analytic models of the international diffusion of consumer technology. Technological Forecasting and Social Change. 2015;100:330-43.

10. Schieritz N, Grobler A, editors. Emergent structures in supply chains-a study integrating agent-based and system dynamics modeling. In 36th Annual Hawaii International Conference on System Sciences, 2003 proceedings of the; 2003 2003: IEEE.

11. Verburg PH, Overmars KP. Combining top-down and bottom-up dynamics in land use modeling: exploring the future of abandoned farmlands in Europe with the Dyna-CLUE model. Landscape ecology. 2009;24(9):1167.

12. Chaim RM, Streit RE. Pension funds governance: combining SD, Agent based modeling and fuzzy logic to address dynamic asset and liability management (ALM) problem. In Proceedings of the 26th International Conference of the System Dynamics Society; 2008: Citeseer; 2008.

13. Dubiel B, Tsimhoni O, editors. Integrating agent based modeling into a discrete event simulation. Proceedings of the Winter Simulation Conference; 2005 2005: IEEE.
