# Supplementary material for: Application of decision analytical models to diabetes in low- and middle-income countries: a systematic review
Source: BMC Health Serv Res. 2022 Nov 23;22:1397. doi: 10.1186/s12913-022-08820-7 (PMC9684986; doi:10.1186/s12913-022-08820-7)
Supplement: Supplementary file 3 — Additional file 3. Characteristics of studies excluded during full text review. [file 12913_2022_8820_MOESM3_ESM.docx]

**Additional file 3**

Characteristics of studies excluded during full text review

| **Study** | **Reason for exclusion** |
| --- | --- |
| Basra et al. (1) | Modelled a sub-population in New Bedford, Massachusetts, USA |
| Chen et al. (2) | Modelled a sub-population in the United States |
| Mash et al. (3) | Modelled cardiovascular disease; the diabetes intervention was evaluated in a cluster randomised trial not in the model. |
| Nagy et al. (4) | Modelled a sub-population in Hungary, an upper-middle-income country |
| Wang et al. (5) | Article published in Chinese |
| Zhou et al. (6) | Baseline model data obtained from the Wisconsin Epidemiologic Study of Diabetic Retinopathy, a population-based study in southern Wisconsin, US |

Reference:

1. Basra K, Fabian MP, Holberger RR, French R, Levy JI. Community-Engaged Modeling of Geographic and Demographic Patterns of Multiple Public Health Risk Factors. Int J Environ Res Public Health. 2017;14(7).

2. Chen F, Jasik CB, Dall TM, Siego CV. Impact of a Digitally Enhanced Diabetes Self-Management Program on Glycemia and Medical Costs. Sci Diabetes Self Manag Care. 2022;48(4):258-69.

3. Mash R, Kroukamp R, Gaziano T, Levitt N. Cost-effectiveness of a diabetes group education program delivered by health promoters with a guiding style in underserved communities in Cape Town, South Africa. Patient Educ Couns. 2015;98(5):622-6.

4. Nagy B, Zsólyom A, Nagyjánosi L, Merész G, Steiner T, Papp E, et al. Cost-effectiveness of a risk-based secondary screening programme of type 2 diabetes. Diabetes Metab Res Rev. 2016;32(7):710-29.

5. Wang JM, Liu QP, Zhang ML, Gong C, Liu SD, Chen WY, et al. [Effectiveness of different screening strategies for type 2 diabete on preventing cardiovascular diseases in a community-based Chinese population using a decision-analytic Markov model]. Beijing Da Xue Xue Bao Yi Xue Ban. 2022;54(3):450-7.

6. Zhou H, Isaman DJM, Messinger S, Brown MB, Klein R, Brandle M, et al. A computer simulation model of diabetes progression, quality of life, and cost. Diabetes care. 2005;28(12):2856â63.
